# Insecticide susceptibility of *Anopheles gambiae s.l* and identification of some resistance mechanisms in Kwilu Province in the Democratic Republic of Congo

**DOI:** 10.11604/pamj.2020.37.79.18635

**Published:** 2020-09-21

**Authors:** Emery Metelo Matubi, Gillon Ilombe Kaounga, Josue Zanga, Guillaume Binene Mbuku, Jean Nguya Kalemba Maniania, Basimike Mulenda, Jonas Nagahuedi Mbongu Sodi, Jean Jacques Muyembe Tamfum, Paul Masiangi

**Affiliations:** 1 Institut National de Recherche Biomédicale de Kinshasa, Kinshasa, République Démocratique du Congo,; 2 Faculté de Médecine, Université de Bandundu, Bandundu-Ville, Bandundu, République Démocratique du Congo,; 3 Faculté des Sciences, Département de Biologie, Unité de Recherche Entomologique, Université de Kinshasa, Kinshasa, République Démocratique du Congo,; 4 Clinical Pharmacology Unit, University of Kinshasa, Kinshasa, Democratic Republic of Congo,; 5 Faculté de Médecine, Université de Kinshasa, République Démocratique du Congo,; 6 International Centre of Insect Physiology and Ecology, Nairobi, Kenya,; 7 Faculty of Medicine, Université Protestante au Congo, Kinshasa, République Démocratique du Congo

**Keywords:** *Anopheles gambiae*, insecticide resistance, Kwilu, Democratic Republic of the Congo

## Abstract

**Introduction:**

the control of the mosquito malaria vectors by the National Malaria Control Programme of the Democratic Republic of Congo (DRC) relies mainly on the use of long-lasting insecticide-treated nets (LLINs). However, the widespread emergence of resistance to pyrethroids is jeopardizing this control strategy. The objective of this study is to determine the status and resistance mechanisms involved in Anopheles gambiae s.l. population of DRC.

**Methods:**

pre-imaginal stages of An. gambiae s.l. were collected and standard WHO bioassays were performed on adult An. gambiae s.l. reared in the laboratory from larvae collected from different sites in the study area. The bioassays with the synergist PBO were also performed to determine the likely implication of oxydases in the resistance. The alleles of knock down resistance (Kdr) gene and species of anopheles were determined by PCR-RLFP.

**Results:**

all Anopheles mosquitoes tested belonged to the Anopheles gambiae complex. An. Gambiae (69.6%) was predominant, followed by An. Coluzzii (25.6%) and (4.8%) hybrids (An. gambiae/ An. coluzzii). Bioassays showed phenotypic resistance to the main insecticides used in the region, notably pyrethroids (deltamethrin, permethrin) and organochlorine (DDT). Only bendiocarb caused 100% mortality. Metabolic resistance involving oxidase enzymes was also detected using the synergist PBO after exposure to deltamethrin. The L1014F allele frequency of Kdr gene was detected in samples collected from all sites at varying frequencies (0.61-1.0).

**Conclusion:**

this study brings additional information on malaria vectors resistance to insecticides. It has shown cross-resistance to DDT and pyrethroids as well as the presence of Kdr gene. PBO significantly improved the effectiveness of deltamethrin. The results of this study can be helpful to policy makers in decision making for vector control programmes in the region.

## Introduction

Despite years of fighting againt malaria vectors, the disease continues to be the leading cause of morbidity and mortality in sub-Saharan African countries [1]. In 2017, 219 million people worldwide suffered from malaria [1] with an estimated 435,000 deaths. Children under 5 years were the most vulnerable and accounted for 61% of all deaths. Nearly 438,000 children and adults die every year from this disease despite the existence of effective preventive and curative measures used in endemic areas [1]. With about 35% of malaria mortality, The Democratic Republic of Congo and Nigeria are the most affected countries worldwide [1]. In DRC, 47% of hospital visits are due to endemic malaria [2,3]. Significant advances in malaria control have been achieved over the past decade, largely to the mass coverage of anti-vector insecticide-based interventions, such as long-lasting insecticide treated nets (LLINs) and indoor residual spraying (IRS) [1,3,4]. LLINs are one of the main malaria control tools recommended by the WHO and adopted by the National Malaria Control Program (NMCP) in the DRC [3,4]. Several studies have demonstrated their effectiveness on large-scale [3-6] and subsequent reduction in morbidity by 50% and overall mortality by 20-30% in children under 5 years [7-10]. Thus, it is of paramount importance in promoting LLINs as core component of NMCP in fighting malaria in DR Congo. Although pyrethroids are the only recommended insecticides for the impregnation of mosquito nets [11-14], several studies have shown that combination of mosquito net and pyrethroids constitute an effective barrier against malaria vectors [15-17].

Despite this large-scale promotion, the disease still remains endemic in the country with a prevalence of 32% [2,3]. Mass distribution of LLINs has prompted resistance of malaria vectors to conventional insecticides [18]. According to the PMI entomological surveillance report and the 2013-2015 strategic plan for malaria control, resistance of anopheline to insecticides is on the increase in the DRC [19-21]. For example, studies carried out in the cities of Kingasani, Bolenge, Kimpese and Katana, have reported an increase of resistance in *An. gambiae s.l*. to pyrethroids and other classes of insecticides such as DDT [19]. In this study, the mechanism of resistance was attributed to the *Kdr* mutation [19]. In addition to development of mosquito resistance to pyrethroids, large-scale use of pyrethroid-impregnated mosquito nets has also resulted in vector behaviour changes such as biting earlier than usual [22]. It is important to note that mosquito nets impregnated with insecticides distributed in Kwilu were of the brand DAWA 2.0 plus [3]. There is the need, therefore, to investigate the real impact of the resistance on the vector control in addition to regular monitoring of the susceptibility of vectors to these insecticides and understanding the mechanisms of this resistance [10,12]. Since studies on the resistance of malaria vectors are lacking in some areas of the DRC, the present study aims at investigating the resistance of malaria vectors in the province of Kwilu.

## Methods

**Figure 1:**
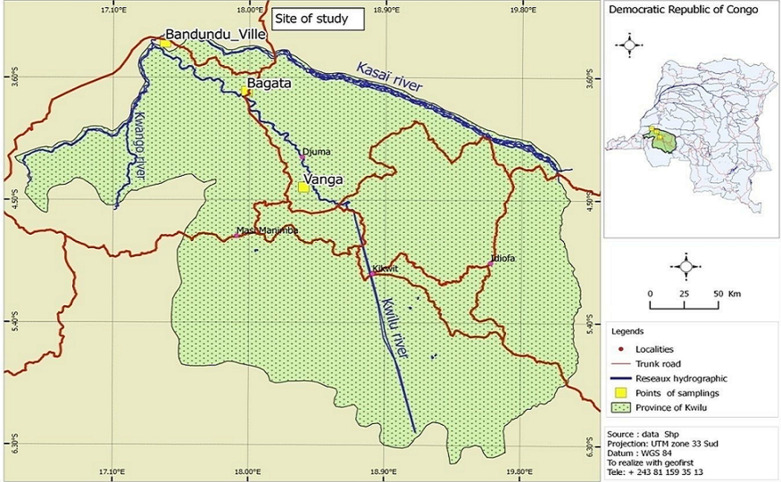
map of Bandundu-city, Bagata and Vanga capture sites in Kwilu, DRC

**Study site:** the study was conducted in 3 districts (Bagata, Vanga and Bandundu-city) in Kwilu Province (former Bandundu), DR Congo, from October 1^st^, 2015 to February 1^st^, 2016. The province is situated between latitude 3°21'05S, longitude 17°22'43''W, 324m above sea level. The province is characterized by two types of vegetation: the forest along rivers and savannas [2,23] (Figure 1). Bandundu-city is surrounded by rivers Kwilu and Kwango at the junction with Kasai, in addition to several streams, thereby making it swampy. The city is made of clay-limestone and hydromorphic soils. The total area is 291km^2^ with a population of approximately 285,411 at the density of 980.79 inhabitants/km^2^. Bagata, 3°43'825''S, 18°50'0E, 400m above sea level. It is a rural health zone located on the left bank of the river Kwilu, 100km away from Bandundu-city. It has an area of 17,776km^2^ and a population of approx. 652,631 inhabitants with a density of 34 in habitants/km^2^. Several streams flow on sandy-clay soils [2,23,24]. Vanga, 15°10'S, 10°9'E, 600m above sea level, 2.600km^2^ area, population of 209.136 inhabitants with a density of 77 inhabitants/km^2^; it is a health zone located on the right bank of the river Kwilu. The soil is sandy-clay.

The province of Kwilu is characterized by a humid tropical climate with two well-marked seasons: the rainy season characterized by mad rainfall and constant heat throughout the year, and a dry season of 4 months [23,24]. The average annual temperature is 26.9°C, the annual rainfall is 800 to 1500mm and the average annual humidity is 77% [24]. The economic activities are organized around the ports with ships sailing between Kikwit and Kinshasa in addition to road transport. The agro-pastoral activity makes the province the breadbasket of the capital, Kinshasa, supplying food and raw materials (palm oil, cassava, maize, peanuts, fish, tobacco) [2,23]. All these sites have been transformed by anthropological activities such as fishing and agriculture (ponds, horticulture, e.t.c.) in addition to livestock and poultry. Houses are made of earth walls and roofs made of corrugated iron or straw. Such houses allow entry-exit of mosquitoes through eaves. These eco-climatic conditions all these conditions are favourable to the development of anopheles vectors of malaria [2,23].

**Mosquito collection:** larvae and nymphs of *An. gambiae s.l*. were collected at different sites identified in each study areas (Bandundu-city, Bagata and Vanga). The collected specimens were kept in tanks with water drawn from the same sites and maintained in insectariums at 25-27°C and relative humidity of 78-80% [24,25].

**WHO´s susceptibility bioassays:** WHO susceptibility tests were performed by selecting and subjecting 2-5-day old females which emerged from larvae and pupae collected from the breeding sites, according to the WHO protocol [25]. Impregnated papers of three approved insecticide classes were used at diagnostic dose: pyrethroids (deltamethrin 0.05%, permethrin 0.75%), carbamate (bendiocarb 0.1%) and organochlorine (DDT 4%). Approximately 100 mosquitoes (four replicates of 25 mosquitoes) were used per test. Control mosquitoes were exposed to untreated papers. The number of *An. gambiae s.l* knocked down at, 3'5'10'15'20'25'30'35'40'45'50'55'60' was recorded during the exposure time while mortality was recorded after 24h [25]. Kdt50 and Kdt95, which represent the shock times after which 50% and 95% of *An. gambiae s.l*. were paralyzed, were determined only for the pyrethrinoid [25]. The efficacy of these compounds was compared with that of DDT and bendiocarb to determine whether or not there was cross-resistance between the three chemical insecticide families. Results were interpreted according to WHO criteria: susceptible (S), all *An. gambiae s.l*. whose mortality 24 hours after insecticide contact was 98-100%; resistant (R) if the mortality of *An. gambiae s.l*. is less than 90%; probable resistant (RP), if the mortality of *An. gambiae s.l*. is between 90 and 97% [25].

**WHO synergist papers:** *An. gambiae s.l*. were pre-exposed to 5% PBO-impregnated paper. WHO´s susceptibility bioassays with synergist PBO (an inhibitor of monooxygenases) were carried out to assess the implication of detoxifying enzymes in the production of resistant phenotypes. Adult female mosquitoes were exposed for 1 h to 5% PBO impregnated papers in batches of 20-25 mosquitoes. PBO was used only for pyrethroids (deltamethrin and permethrin) because of its role on cytochrome P450 monooxygenase, implicated in the resistance of anopheles to these insecticides [26-28].

**Identification of mosquitoes:** adult mosquitoes were identified based on morphological characteristics according to the dichotomous key of Gilles and De Meillon [29] and were individually stored in 1.5ml eppendorfs tubes containing silica gel for molecular analysis. A total of 150 *An. gambiae s.l*. (resistant and susceptible) (50 per site) were randomly selected from the three sites for molecular analysis.

**Detection of the *Kdr* gene (L1014F):** the genomic DNA of each mosquito was extracted and amplified according to the protocol of Fanello *et al*. [30]. The PCR for the detection of *Kdr* mutations was carried out according to the protocol described by Martinez-Torres *et al*. [31]. The allele frequency of *Kdr* genes was calculated based on the genetic formula of Hardy-Weinberg: F(*Kdr*)=2NRR+NRS/2(NSS+NRS+NRR) [32].

**Statistical analysis:** the required time (in minutes) to obtain 50% and 95% knockdown mosquitoes (Kdt50 and Kdt95) was calculated using log-probit regressions; Polo Plus version 1.0 software [33], according to WHO protocol. The Chi-square test was used to compare the mortality of *An. gambiae s.l*. between insecticide tests alone and after pre-exposure to synergist (PBO) at the significance level of 0.05. The effect of synergists was calculated with effective values above 10%. The allelic frequency of *Kdr* genes was calculated and the correlation was established between the species and the distribution of the *Kdr* genes according to the sites [32].

## Results

Susceptibility tests carried out on wild populations of *An. gambiae s.l*. from the three sites of Kwilu in DRC made it possible to better understand the levels of tolerance of this vector to the two pyrethroids (permethrin and deltamethrin) commonly used in public health for the impregnation of mosquito nets. This insecticide efficacy was measured based on their knock-down effect and mortality after 24 hours of observation. The results are shown in Table 1. The knock down effect of insecticides on *An. gambiae s.l*. varied according to the type of insecticide and the site of capture. The Kdt50 and Kdt95 were observed only with deltamethrin (Table 1) although they took longer. For example, 50% of anopheles were shocked between 25.1-47.9 minutes while 95% were shocked after 53 minutes. This was observed only in Bagata and Vanga (Table 1). No knock down effect (Kdt95) were observed in Bandundu-city. Only Bendiocarb was very active in anopheles with 100% mortality 24 hours after exposure. DDT, deltamethrin and permethrin were the least active insecticides in all study sites. Pre-exposure to synergists resulted in the reduction of shock time. Kdt50 and Kdt95 values were observed with deltamethrin in all the sites but varied according to site. Kdt50 values were short (16.5 minutes) with deltamethrin in Bandundu-city and longer with permethrin (54 minutes) in Vanga following exposure. On the other hand, Kdt95 was not observed with permethrin in all the sites (Table 1).

**Table 1:** mortality of *Anopheles gambiae s.l* 24h post-exposure to insecticide at three sites of Kwilu Province

Sites	Insecticides	N	KdT50 (minute)	KdT95 (minute)	Mortality 24h	Status
Bandundu-ville	Deltaméthrine 0.05%	100	43.8 (39.9-47.9)	No effect	52	R
	Deltaméthrine 0.05% + PBO 4%	100	16,5 (15-17.8)	38.5 (35-43)	98	S
	Permethrine 0.75%	100	No effect	No effect	17	R
	Permethrine 0.75% + PBO 4%	100	No effect	No effect	88	R
	Bendiocarb 0.1%	100	-	-	100	S
	DDT 4%	100	No effect	No effect	2	R
Bagata	Deltaméthrine 0.05%	100	26.8 (25.1-28.4)	55.3 (50.8-61.3)	81	R
	Deltaméthrine 0.05% + PBO 4%	100	16.5 (15-17.8)	38.5 (35-43)	100	S
	Permethrine 0.75%	100	No effect	No effect	31	R
	Permethrine 0.75% + PBO 4%	100	42.3 (39.4-45.3)	No effect	100	S
	Bendiocarb 0.1%	100	-	-	100	S
	DDT 4%	100	No effect	No effect	5	R
Vanga	Deltaméthrine 0.05%	80	32.2 (32-34.4)	53.6 (51-57)	64	R
	Deltaméthrine 0.05% + PBO 4%	80	22.5 (20-25)	32 (30-34)	100	S
	Permethrine 0.75%	80	No effect	No effect	30	R
	Permethrine 0.75% + PBO 4%	80	54 (51-59)	No effect	91	RP
	Bendiocarb 0.1%	80	-	-	100	S
	DDT 4%	80	No effect	No effect	21.3	R

**Determination of some resistance mechanisms of *An. gambiae s.l*.:** the frequency of allelic gene *Kdr* L1014F from West and Central Africa was determined on anopheles and the oxidases were indirectly identified by the use of synergist PBO (piperonil butoxide inhibitor). The effect of PBO was observed in all the sites with anopheles treated with deltamethrin and partially with the ones treated with permethrin. Mortality of mosquitoes treated with deltamethrin alone using OMS test was 64, 81 and 52% for Vanga, Bagata and Bandundu-city, respectively. However, mortality increased significantly (χ^2^= 35.5, p <0.001) to 100 in Vanga, Bagata and 98% in Bandundu-city following pre-exposure to PBO (Table 2). These results suggest the involvement of oxydases in resistance mechanism of vector populations to deltamethrin. On the other hand, a slight increase in mortality was observed with permethrin. Mortality of mosquitoes treated with permethrin alone was 30, 31 and 17% for Vanga, Bagata and Bandundu-city, respectively. Mortality increased significantly (χ^2^= 93.1, p<0.001) following pre-exposure to PBO. Indeed, mortality reached 100% for Vanga and Bagata and 88 % for Bandundu-city (Table 1).

**Table 2:** involvement of P450 mechanisms in pyrethroid resistance in Bandundu-city, Bagata and Vanga as determined by PBO synergist assays

Sites	Insecticides	N	Mortality 24 h	Interpretation
Bandundu-ville	Deltaméthrine 0.05%	100	52	Full involvement of P450 oxidase mechanisms
	Deltaméthrine 0.05% + PBO 4%	100	98	
	Permethrine 0.75%	100	17	Partial involvement of P450 oxidase mechanisms
	Permethrine 0.75% + PBO 4%	100	88	
Bagata	Deltaméthrine 0.05%	100	81	Full involvement of P450 oxidase mechanisms
	Deltaméthrine 0.05% + PBO 4%	100	100	
	Permethrine 0.75%	100	31	Full involvement of P450 oxidase mechanisms
	Permethrine 0.75% + PBO 4%	100	100	
Vanga	Deltaméthrine 0.05%	80	64	Full involvement of P450 oxidase mechanisms
	Deltaméthrine 0.05% + PBO 4%	80	100	
	Permethrine 0.75%	80	30	Partial involvement of P450 oxidase mechanisms
	Permethrine 0.75% + PBO 4%	80	91.3	

Out of the 150 anopheline samples selected for PCR, 9 individual mosquitoes were spoiled and could not be processed. The results of the remaining 141 samples analysed by PCR-RFPL are presented in Table 3. The following anopheles species were identified: 61.7% (87/141) was *An. gambiae*, 22.7% (32/141) *An. coluzzii*, 4.3% (6/141) hybrids and 11.3% (16/141) non-*gambiae s.l*. The *Kdr* gene was more weight-bearing in *An. gambiae*, 31.2% (44/141) and least´s in at *An. coluzzii*, 16% (23/141). The allelic frequency of this gene was variable depending on species and site. The frequency for *An. gambiae* was 0.76, 0.61 and 0.63 for Bandundu-city, Bagata and Vanga, respectively, while it was 0.90, 0.77 and 0.83, respectively for *An. coluzzii*. There was no significant difference between the Kdr gene distribution and the anopheles species (*An. gambiae* and *An. coluzzii*) at the threshold α=0.05; χ2=3.3; P=0.071.

**Table 3:** target site *kdr* mutation in Bandundu-city, Bagata and Vanga

	Bandundu-city				Bagata				Vanga			
	RR	Rs	ss	F(*kdr*)	RR	Rs	Ss	F(*kdr*)	RR	Rs	ss	F(*kdr*)
*An. gambiae s.s*.	24	16	2	0.76	8	7	4	0.61	12	9	5	0.63
*An. Coluzzii*	4	1	-	0.90	10	1	1	0.87	9	5	1	0.77
*An. gambiae ss/coluzzii* hybrid	-	-	-	-	1	-	-	1.0	5	-	-	1.0
Other species	-	1	-	0.50	7	1	-	0.94	5	2	-	0.86

## Discussion

Vector control is an essential component for malaria control and elimination [1, 5]. The ability of vectors to transmit parasites and their vulnerability to anti-vector measures varies according to mosquito species and is influenced by local environmental factors. Thus, it is important that vector control be carried out on the basis of local epidemiological and entomological data [1,4].

**Anopheles sibling species:** two Anopheles species of *Anopheles gambiae* complex were identified, *An. Gambiae* and *An. coluzzii*, among which there were hybrids *An. gambiae/coluzzii. An. Gambiae* was majority in Bandundu-city with 84% and minority in Bagata with 38%. *An. Coluzzii* was present in all the three sites in different proportions: 10, 24 and 30% in Bandundu-city, Bagata and Vanga, respectevely. These observations show that these species are sympatric and their distribution varies, with predominance of An. gambiae at 69.6%. These results corroborate the findings of Wat'senga who reported 64.1% *An. Gambiae* and 35.9% *An. Coluzzii* in Kinshasa and of Simard in northern Cameroon, where 93.6% *An. Gambiae* and 6.4% *An. Coluzzii* were recorded. No hybrid was reported by the latter author and Carnavale in Angola [34-36]. Both species have been sympathetic and their hybrids (12-6%) vary with site and harvest period [36,37]. These results are, however, in contradiction with the ones from Bobanga *et al*. (2016) who reported 100% *An. Coluzzii* in Bandundu-city and in Vanga (100% *An. Coluzzii* in this two site) [38]. The difference could be explained by the sample size and methodology used of in those studies. They are opposed to those found by Konan *et al*. (coluzzii = 98%, S = 2%) [39,40]. This difference in the distribution may probably be related to the ecology of the vector.

**Figure 2:**
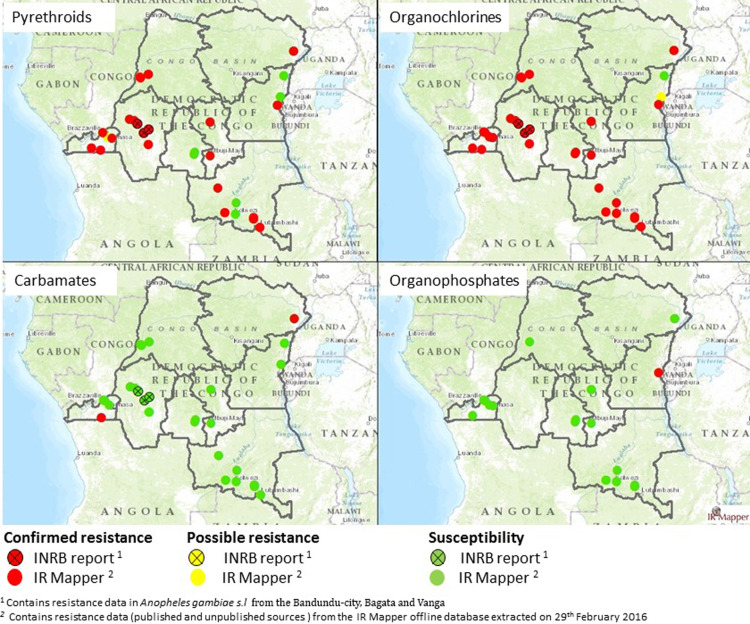
status of *Anopheles gambiae s.l*. against insecticides at study sites in DRC

**Resistance to pyrethroids:** with exception to bendiocarb that caused 100% anopheline mortality, all the insecticides tested in the present study were ineffective to the *An. gambiae s.l*. In Kwilu, *An. gambaie s.l*. were found to be resistant to pyrethroids (deltamethrin and permethrin) and DDT. The mortality of anophelines to insecticides varied according to the site. It was limited to deltamethrin, 52% to Bandundu-city, 81% to Bagata and 64% to Vanga, which reduced the effectiveness of this product. Anopheles mosquitoes were also resistant to the dose of all sites, Bandundu-city, Bagata and Vanga with mortality rates of 17%, 31% and 30% respectively. Our results corroborate those of Basilua, Riveron and Watsenga in the DRC (Figure 2) [19,21,41] and other workers in sub-Saharan Africa [42-44]. This resistance raises fundamental and operational problems, in particular, to which extent anopheles behavior is modified and whether this resistance to pyrethroids is accompanied with significant decrease in the effectiveness of these products [18,41]. Persistence of malaria endemicity and the high number of *An. Gambiae* infected (sporozoite rate 5.6%) following the study of Metelo *et al* in Bandundu-city can address this preoccupation [45]. Kdt50 and Kdt95 were highly varied according to the site and the type of insecticide. For instance, Kdt50 and Kdt95 were not observed with DDT and permethrin did not have Kdt50 and Kdt95 effect on anopheles at all the sites.

Kdt50 was delayed with deltamethrin and varied according to the site, Bandundu-city, Bagata and Vanga (45, 22.5 and 32.5 minutes). This absence of shock effect and its late appearance confirms the occurrence of the observed resistance. This cross resistance to pyrethroids (deltamethrin and permethrin) and DDT had already been reported in several sites in the DRC as shown by IR Mapper. This illustrates the risk of emergence and spread of resistance in the DRC (Figure 2). It can be attributed to a likely inheritance of DDT resistance reported in other sites in the country. These results corroborate those obtained by Basilua in the DRC and other authors in the Afro-tropical zones [12,19,21,46,47]. Considering the current dependence of vector control based on the use of insecticides and the increase in resistance, there is the need to develop new and sustainable control strategies. Management of this resistance to insecticides is complex, partly because it can take various forms (metabolic and genetic resistance) [4,18,27]. To determine the mechanism of resistance, *An. gambiae s.l*. from the three different study sites were pre-exposed to the synergist PBO (an inhibitor of monooxygenases). As a result, the susceptibility of mosquito populations to pyrethroids (permethrin and deltamethrin) increased significantly.

Oxydases were implicated in the metabolic resistance mechanism of anopheles tested with deltamethrin in all the sites. Except in Bandundu-city where to observe the partial involvement of P450 oxidase mechanisms for permethrin. These observations corroborate those of other authors in the afro-tropical zones [9,13,17]. In addition, this resistance was related to the *Kdr* genes with allele frequency L1014F very high in Bagata, 68% in Bandundu-city and 58% in Vanga. Similar results were reported by Basilua [7,19,41,48]. No significant difference was observed between *An. Coluzzii* and *An. Gambiae* in the expression of *Kdr* resistance genes and the detoxification of pyrethroids by oxidases in all the studied sites. These results are contrary to those reported by Yahouedo in Benin where a significant difference was observed [7,42]. This strong resistance of the *An. gambiae s.l*. to deltamethrin, permethrin and DDT in the province of Kwilu could be linked to the intensive use of insecticides in agriculture (palm oil plantations, cotton, tobacco, e.t.c.) [2,23]. Similar observations were reported in Burkina Faso, Cote d'Ivoire in agricultural settings and other afro-tropical zones [9,12,49]. The allelic frequency of the *Kdr* gene observed varied according to the species and sites. The high allelic frequencies observed in *An. Gambiae* and *An. Coluzzii* could be explained by the excessive use of insecticides in the households due to the nuisance caused by the insects in the country. Ultimately, new compounds will be needed to manage resistance and delay the spread of resistance and preserve insecticide susceptibility, at least until new classes or molecules are available. To be effective, local strategies must be adapted to the type of resistance encountered in Kwilu Province.

## Conclusion

*An. gambiae s.l* was resistant to pyrethroids and DDT (cross-resistance) in all the sites. An increase in susceptibility of *An. gambiae s.l* to pyrethroids was observed after pre-exposure to PBO 5%. This resistance was largely metabolic in origin, related to P450 mono-oxygenases and the presence of high-level 1014F allele *Kdr* gene was detected. Since there is resistance emergence to the only one class of insecticide (pyrethroids) recommended for the impregnation of mosquito nets in mosquito vectors of Kwilu. There is a major concern over their repellent and lethal efficacy. Hence, there is the need to consider a new alternative to curb the emergence of this resistance and maintain the gains achieved.

### What is known about this topic

- The profile of the sensitivity of anopheles to insecticides is partially documented in the DRC;
- The mechanisms of genetic and metabolic resistance are partially documented and fragmentary in Africa and the DRC;
- Since mutation is a selection, the progressive development of resistance to the various insecticides used in public health is influenced by both anthropogenic and environmental factors.

### What this study adds

- The resistance of the population of An. gambiae s.l. from Kwilu observed to pyrethroids (permethrin and deltamethrin) was significantly improved by the use of PBO;
- The genes Kdr L1014F and oxydase have been identified as the main resistance mechanisms used by An. gambiae s.l. against insecticides used in public health in the Kwilu region;
- The present study aims to fill the lack of data on the resistance status of An. gambiae s.l. towards insecticides in Kwilu.
